# An alternative approach to combination vaccines: intradermal administration of isolated components for control of anthrax, botulism, plague and staphylococcal toxic shock

**DOI:** 10.1186/1476-8518-6-5

**Published:** 2008-09-03

**Authors:** Garry L Morefield, Ralph F Tammariello, Bret K Purcell, Patricia L Worsham, Jennifer Chapman, Leonard A Smith, Jason B Alarcon, John A Mikszta, Robert G Ulrich

**Affiliations:** 1 Department of Immunology, Army Medical Research Institute of Infectious Diseases, Frederick, MD, USA; 2 Molecular Biology, Army Medical Research Institute of Infectious Diseases, Frederick, MD, USA; 3 Bacteriology, Army Medical Research Institute of Infectious Diseases, Frederick, MD, USA; 4 Pathology Divisions, Army Medical Research Institute of Infectious Diseases, Frederick, MD, USA; 5 Becton Dickinson Technologies, Research Triangle Park, NC, USA

## Abstract

**Background:**

Combination vaccines reduce the total number of injections required for each component administered separately and generally provide the same level of disease protection. Yet, physical, chemical, and biological interactions between vaccine components are often detrimental to vaccine safety or efficacy.

**Methods:**

As a possible alternative to combination vaccines, we used specially designed microneedles to inject rhesus macaques with four separate recombinant protein vaccines for anthrax, botulism, plague and staphylococcal toxic shock next to each other just below the surface of the skin, thus avoiding potentially incompatible vaccine mixtures.

**Results:**

The intradermally-administered vaccines retained potent antibody responses and were well- tolerated by rhesus macaques. Based on tracking of the adjuvant, the vaccines were transported from the dermis to draining lymph nodes by antigen-presenting cells. Vaccinated primates were completely protected from an otherwise lethal aerosol challenge by *Bacillus anthracis *spores, botulinum neurotoxin A, or staphylococcal enterotoxin B.

**Conclusion:**

Our results demonstrated that the physical separation of vaccines both in the syringe and at the site of administration did not adversely affect the biological activity of each component.

The vaccination method we describe may be scalable to include a greater number of antigens, while avoiding the physical and chemical incompatibilities encountered by combining multiple vaccines together in one product.

## Background

Vaccination compliance will predictably become a significant concern as current schedules approach the limit of public acceptance [1] and new vaccines become available. The development of combination vaccines is a common practice that addresses the concern of repeated visits to the clinic by reducing the total number of injections required compared with administration schedules for the monovalent vaccines. Yet, physical, chemical, and biological interactions between the components of combination vaccines must be considered to avoid detrimental effects on safety or efficacy. For example, when the *Haemophilus influenzae *type b (Hib) vaccine was combined with diphtheria, tetanus, and acellular pertussis vaccine, a decrease in antibody titer for the Hib vaccine was observed [2]. Thus, there is a need to develop new approaches for delivery of multiple vaccines.

We evaluated delivery of multiple vaccines intradermally (i.d.) to physically isolate each component, thus directly preventing formulation incompatibilities prior to administration. The physiological fate of vaccines administered i.d. is not known. However, vaccination by microneedles [3] permits verification of the physical deposition into the skin while intramuscular (i.m.) injection sites are inaccessible for direct observation. Further, i.d. vaccination using microneedles is less painful [3] than i.m. injection by conventional needles and provides an increased immune response with a lower amount of vaccine than that required by intramuscular (i.m.) methods [4,5]. The greater efficacy resulting from i.d. vaccination may permit the administration of an increased number of vaccines compared to i.m. because a smaller volume is required for delivery.

The pre-clinical phase of vaccine development traditionally focuses on a single disease of concern, often targeting a protein that is critical to pathology. Because emerging infectious diseases and agents of concern to biodefense contribute substantially to the burden of new vaccines, we specifically examined vaccines for anthrax, botulism, toxic-shock syndrome, and plague. The following is a brief description of the diseases and vaccines that were developed for prevention.

*Bacillus anthracis*, the etiological agent of anthrax, produces binary toxins [6-9] comprised of protective antigen (PA) combined with lethal factor (LF) or edema factor (EF). The vaccine employed in our study was a recombinant form of PA (rPA) that was previously shown to protect rhesus macaques from aerosol challenge with *B. anthracis *spores [10,11]. Antibodies that neutralize PA block the transport of LF and EF to the cytosol, thereby blocking cell death induced by the toxins. Botulinum neurotoxin type A (BoNT/A) causes botulism by blocking the release of acetylcholine at the neuromuscular junction [12]. A recombinant C fragment vaccine of botulinum neurotoxin type A [BoNT/A(H_c_)] was developed that does not possess the toxic properties of the wild-type protein [13]. In previous studies, the BoNT/A(H_c_) was shown to be effective at protecting vaccinated mice against challenge with the wild-type toxin [13]. Antibodies that prevent botulism are presumed to inhibit binding of the toxin to neurons and thereby impede entry of the toxin into the cell. Staphylococcal enterotoxin B (SEB) is a virulence factor expressed by most isolates of the common human pathogen *Staphylococcus aureus *[14,15]. Secreted SEB binds and cross-links class II molecules of the major histocompatibility complex expressed on antigen-presenting cells to the antigen receptors on T cells, leading to potent activation of the immune system. Life-threatening toxic shock syndrome may result from the rapid release of high levels of IFN-γ, IL-6, TNF-α and other cytokines in response to SEB. The recombinant SEB vaccine (STEBVax) contains three site-specific mutations that collectively alter key protein surfaces, leading to loss of receptor binding and superantigen activity [16]. This vaccine was shown in previous studies to protect rhesus macaques from aerosol challenge with SEB [17] and protection from toxic shock in vaccinated monkeys correlated with SEB neutralization by antibodies [17]. We also examined an experimental plague vaccine (F1-V) consisting of a recombinant fusion protein of the bacterial antigens CaF1 and LcrV, previously shown to protect mice against plague [18,19]. The bubonic form of plague results from *Yersinia pestis *injected into the skin by the bite of infected fleas and is characterized by acute painful swelling of regional lymph nodes. Progression to septicemic or secondary pneumonic plague may also ensue. Primary pneumonic plague may also occur by transfer of bacteria through aerosols produced by coughing. Although mouse data are available [18,19], there are no reports that address protection of non-human primates that were vaccinated with F1-V and challenge with *Y. pestis*. However, we included F1-V in our study to increase the complexity of the vaccine combination and because this high-profile product is ultimately intended for human use.

All of the vaccines we investigated were developed independently, using buffers and additives that were potentially incompatible if all antigens were directly mixed due to differences in pH, buffers, and stability profiles. For example, STEBVax was maintained in a glycine buffer of pH 8, while a phosphate buffer of pH 7 was used for rPA. Yet, an advantage associated with the vaccines for anthrax, botulism and staphylococcal toxic shock is that all were previously examined in studies using rhesus macaques [[10,11,17], and unpublished observations], allowing us to measure survival from an otherwise lethal sepsis in the same animal disease model. Although co-formulation may ultimately be achievable for many vaccines, physical separation obviates the need for additional costly studies to re-examine safety, stability, and efficacy. We hypothesized that the physical separation of vaccines both in the syringe and at the site of administration will not adversely affect the biological activity of each component.

## Methods

### Vaccinations

The recombinant botulinum neurotoxin serotype A binding domain BoNT/A(H_c_), SEB vaccine (STEBVax) and the fusion protein of F1 and V antigens (rF1-V) were prepared as previously described [10,13,16,19]. The recombinant protective antigen (rPA) was obtained from List Laboratories (Wako, TX). Each vaccine was combined with AH adjuvant (Superfos Biosector, Kvistgård, Denmark), before administration using previously optimized ratios (unpublished observations) that in all cases resulted in delivery of < 1 mg of elemental aluminum per animal. Rhesus monkeys were obtained from Primate Products, Inc. (Woodside, CA) and quarantined for 30 d before study initiation. Just before vaccination, anesthetized (ketamine/acepromazine) monkeys were shaved on the deltoid/upper arm region or thigh using electric clippers, and the vaccines were administered i.d. on days 0, 28, and 56. On day 0 the vaccines were administered on the left arm, on day 28 the vaccines were administered on the right arm, and on day 56 the vaccines were administered on the left thigh. Vaccinated animals received 5 μg of the BoNT/A(H_c_) vaccine, 150 μg of rF1-V, 50 μg of rPA, and 40 μg of STEBVax. Control animals received injection of AH adjuvant with no antigen. Two 100-μl i.d. injections of each vaccine were administered 2 cm apart with a stainless steel microneedle (1-mm exposed length, 76-μm inner diameter, 178-μm outer diameter) attached to a 1-ml syringe, as previously described [20].

### Serology

Complete blood counts with white blood cell differential counts as well as serum concentrations of IgM and IgG were determined from blood collected on days 14, 42, and 70. Before each blood draw, animals were anesthetized by injection with ketamine/acepromazine. Antigen-specific serum antibody levels were determined by ELISA. Plastic plates (96 well) were coated (1 h, 37°C) with 100 μl/well of 2 μg/ml of BoNT/A(H_c_), rF1-V, rPA, or STEBVax diluted in PBS (pH 7.4) for the sample unknowns, and purified monkey IgM or IgG was serially diluted threefold for the standard curve. The plates were washed three times with PBS/0.1% Tween and blocked (1 h, 37°C) with 0.2% casein/PBS (100 μl/well), washed as above, and then were incubated (1 h, 37°C) with 100 μl of diluted serum samples. Plates were then washed and incubated (1 h, 37°C) with 100 μl/well of goat anti-monkey IgG or goat anti-monkey IgM (1:10,000 dilutions) conjugated to horseradish peroxidase, washed, and developed (30 min, 22°C) with 100 μl of TMB peroxidase substrate (KPL, Gaithersburg, MD). Absorbance was measured at 650 nM and concentrations were determined by comparison to the absorbance of the standard curve.

### Neutralizing antibody assays

For the anthrax toxin neutralization assay, 100 ng/ml LF and 200 ng/ml of PA, both in high-glucose DMEM with 7.5% fetal bovine serum (FBS), were mixed 1:1 with dilutions of sera and incubated for 1 h (37°C) before being added to J774 cells growing on a 96-well plate (63,000 cells/well in high-glucose DMEM, 7.5% FBS). The cells were incubated at 37°C for 4 h and cell viability was determined by ATP content (Vialight HS, Cambrex, Rockland, ME). The endpoint titer was determined as the serum dilution that gave a response three times greater than background. For the SEB neutralization assay, human peripheral blood mononuclear cells were isolated by density gradient centrifugation and added to a 96-well plate (100,000 cells/well in RPMI, 5% fetal calf serum). After plating, cells were allowed to rest for 2 h at 37°C. Dilutions of the test and control sera were prepared and SEB (200 ng/ml) was added to each dilution. Serum dilutions were then incubated for 1 h. at 37°C. The treatments (50 μl/well) were added to the cells and the plates were incubated at 37°C for 60 h. Finally, 1 μCi of [^3^H] thymidine (Sigma, St. Louis, MO) was added to each well, the plates were incubated for 9 h at 37°C, and incorporated radioactivity was measured by liquid scintillation. The antibody titer was determined as the highest serum dilution that significantly inhibited (Student's t-test) SEB-induced proliferation of the monocytes compared to the negative control. For the BoNT/A neutralization assay, dilutions of serum from animals in the BoNT/A challenge groups were mixed with 10 LD_50 _of toxin and incubated for 1 h at room temperature. Each dilution was injected intraperitoneally (IP) into four CD-1 mice. The mice were observed for 4 days and the number of deaths in each group was recorded. The neutralizing antibody titer was determined as the reciprocal of the serum dilution that protected 50% of the mice from intoxication with BoNT/A.

### Aerosol challenge

Animals were split into four separate challenge groups, each containing two controls and six vaccinated monkeys. Each group was challenged with one agent: BoNT/A, Ames strain spores of *B. anthracis*, or SEB, all obtained from USAMRIID. Before challenge, monkeys were anesthetized with ketamine/acepromazine and their breathing rate was determined by plethysmography. For groups challenged with botulinum neurotoxin A (50 LD_50_), *B. anthracis *(200 LD_50_), or SEB (25 LD_50_), each animal was exposed to the agent for 10 min in a head-only exposure chamber. Animals were observed up to two months after challenge. On days 2, 4, and 6 postchallenge, blood was drawn and complete blood counts with white blood cell differential counts were performed on all samples and bacteremia was determined for samples from animals challenged with bacterial agents. Necropsies were performed on animals that did not survive to verify death was a result of exposure to the challenge agent.

### Pathology and necropsy

A necropsy was performed on all animals, either as soon as death occurred from infection or intoxication or after humane euthanasia of terminally ill or moribund animals by established protocols. Samples of spleen, lymph nodes (mandibular, axillary, tracheobronchial, mesenteric), lung, trachea, mediastinum, and haired skin from the vaccine sites from each monkey were collected for histopathology. Additionally, brain tissue was collected from animals that succumbed due to infection with *B. anthracis*. All tissues were immersion-fixed in 10% neutral buffered formalin.

### Histology and immunohistochemistry

Formalin-fixed tissues for histology were trimmed, processed, and embedded in paraffin according to established protocols [21]. Histology sections were cut at 5–6 μm, mounted on glass slides, and stained with hematoxylin & eosin (H&E). Immunohistochemical staining was performed using the Envision+ method (DAKO, Carpinteria, CA). Briefly, sections were deparaffinized in Xyless, rehydrated in graded ethanol, and endogenous peroxidase activity was quenched in a 0.3% hydrogen peroxide/methanol solution for 30 min at room temperature. Slides were washed in distilled water, placed in a Tris-EDTA Buffer (10 mM Tris Base, 1 mM EDTA Solution, 0.05% Tween 20, pH 9.0) and heated in a vegetable steamer for 30 min. Sections were incubated in the primary antibody, rabbit anti-major histocompatibility complex class II polyclonal antibody (RGU, unpublished), diluted 1:500 for 1 h at room temperature. After the primary antibody incubation, sections were washed in PBS and incubated for 30 min with Envision + System HRP (horseradish peroxidase-labeled polymer conjugated to goat anti-rabbit immunoglobulins) at room temperature. Peroxidase activity was developed with 3,3'-diaminobenzidine (DAB), counterstained with hematoxylin, dehydrated, cleared in Xyless, and coverslips were applied with Permount.

### Adjuvant visualization in tissues

Adjuvant was localized in tissue samples by detection of aluminum. Five micrometer sections were prepared from formalin fixed, paraffin-embedded tissue blocks, deparaffinized in Xyless, and rehydrated in graded alcohols. Slides were rinsed in distilled water then pretreated in a 1% aqueous solution of hydrochloric acid for 10 min. After rinsing the slides in distilled water for 5 min, we stained them in a 0.2% alcoholic Morin solution (Sigma, Atlanta, GA) for 10 min. After staining with Morin, the sections were incubated for 2 h at 37°C with a 1:20 dilution of Texas Red phalloidin and approximately 1 μg/ml of Hoechst-33258 (Molecular Probes, Eugene Oregon) in PBS. Sections were rinsed twice in PBS and once in water before coverslips were applied with Vecta Shield mounting medium (Vector Labs, Burlingame, CA).

### Confocal microscopy

Images were collected with a BioRad 2000 MP confocal system attached to a Nikon TE300 inverted microscope fitted with a 60× (1.20 N.A.) water-immersion objective lens. Morin fluorescence was detected with 488 nm laser excitation and a HQ515/30 emission filter. Texas Red phalloidin was imaged with 568 nm laser excitation and an E600LP emission filter. Hoechst dye was visualized with 800 nm 2-photon excitation and a HQ390/70 emission filter. Subsequent contrast enhancement of the resulting images was performed using Adobe PhotoShop software.

### Statistical analysis

Analysis of variance was used to analyze serology data obtained at various time points after vaccine administration to determine if there were any statistical differences within or between the vaccinated and control groups. The data conformed with the assumptions of the test if plots of the residuals revealed no structure. Comparisons of antibody production and lymphocyte proliferation between vaccinated and control animals were performed using Student's t-test. The data conformed to the assumptions of the t-test if the normal probability plot was a straight line. Historical controls were used to increase the statistical power of the experiment. Uniform lethality was observed in more than 15 untreated control Rhesus exposed to the same strain and route of each agent used in the experiment. Efficacy was evaluated using Fishers exact test comparing the treated group to the control group for each agent consisting of 2 experimental controls and 15 historical controls.

## Results

### Intradermal administration of physically separated vaccines

**Figure 1:**
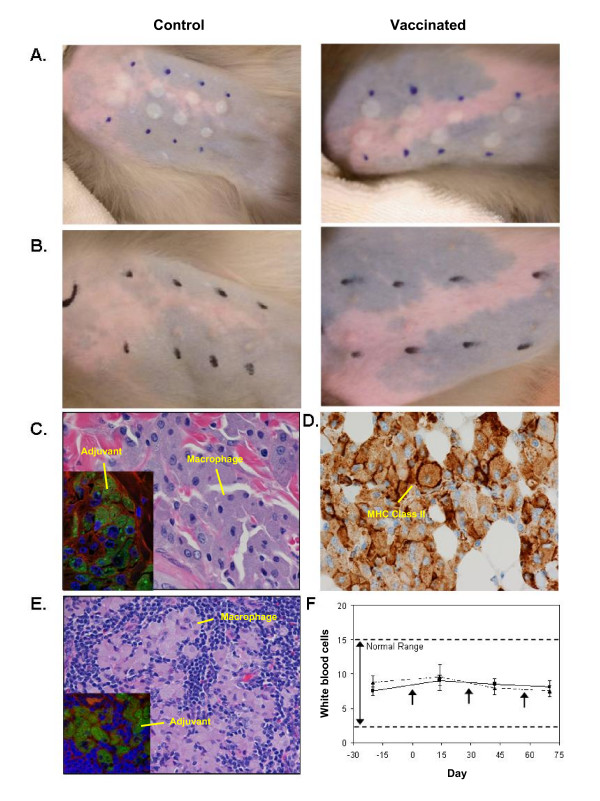
**Intradermal administration of the vaccines for anthrax (rPA), botulism [BoNT/A(H_c_)], plague (rF1-V), and SEB induced toxic-shock (STEBVax)**. A. Rhesus macaque skin immediately after vaccination (two sites, left to right): BoNT/A, rF1-V, rPA, and STEBVax. B. Rhesus macaque skin two months after vaccine administration. Marks are adjacent to injection sites. C. Skin sections (H&E stain) obtained from the vaccine delivery site exhibited epithelioid macrophages and multinucleated giant cells containing adjuvant (inset, green). Phalloidin staining of actin, red; Hoechst staining of DNA, blue. D. Macrophages at the vaccine delivery site exhibited high expression of MHC-II molecules (brown). Anti-MHC Class II immunohistochemistry (brown). E. Epithelioid macrophages (H&E stain) containing adjuvant (inset) were also present in the axillary lymph nodes of vaccinated animals. F. Vaccination did not significantly alter white blood cell counts of vaccinated animals (solid line) compared to control (dashed line). Mean cell counts ± SD of all animals studied.

A simple mixture of the BoNT/A(H_c_), F1-V, rPA and STEBVax as currently formulated resulted in formation of a precipitation and a significant change in pH of the solution (data not shown). Because of these apparent chemical incompatibilities we were not able to examine animals vaccinated with simple mixtures of the vaccines. The vaccines BoNT/A(H_c_), F1-V, rPA and STEBVax were individually administered three times, 28 d apart, by injection into the shaved dermis of the upper arm or thigh of rhesus macaques using stainless steel microneedles that were the approximate diameter of a human hair, as previously reported [18-21]. The subject animals received doses of each vaccine that were independently optimized [11,13,17,19] and adsorbed to aluminum hydroxide adjuvant (AH). Control animals received i.d. injections of AH alone. The pattern of vaccinations consisted of an array of 100-μl injections separated by 2 cm, keeping each vaccine isolated from adjacent administrations (Fig. 1).

No visible indications of discomfort were noted in any animal after vaccination. Slight erythema was evident at sites of second or third vaccinations, suggesting a robust recall immune response. Small raised blebs appeared on the skin at each injection site (Fig. 1A) immediately after vaccine administration, and the sites were only slightly perceptible on the surface of the skin up to 2 months later (Fig 1B). Histology performed on tissue samples obtained from the delivery site showed AH localized within the dermis after administration and a granulomatous response to vaccination in both the controls and vaccinates (Fig. 1C). Numerous phagocytes and multinucleated giant cells were present in the dermis and panniculus at the injection site and the phagocytes contained abundant intracytoplasmic blue-gray granular material (Fig. 1C). Histochemical staining of the tissue with Morin, a dye that is fluorescent green upon chelation of aluminum, demonstrated positive staining of the intracytoplasmic granular material, which verified the presence of aluminum from the vaccine adjuvant (Fig. 1C inset). Immunohistochemical staining of the skin revealed that the phagocytes exhibited expression of MHC-II molecules (Fig. 1D). Examination of tissue from the axillary lymph nodes revealed phagocytes that contained a similar intracytoplasmic granular material as the skin sections (Fig. 1E). As before, staining the tissue with Morin revealed positive, fluorescent intracytoplasmic granules, verifying the material was aluminum from the vaccine adjuvant (Fig. 1E inset). These results suggest that the vaccines were transported from the dermal injection site to the draining lymph nodes.

Several diagnostic parameters were monitored during the study to evaluate the safety of simultaneous administration of multiple vaccines. Vaccine administration did not significantly affect the white blood cell counts of either the controls or vaccinated animals (Fig. 1E). No abnormalities were noted in red blood cell count, platelets, hemoglobin, hematocrit, mean corpuscular volume, mean corpuscular hemoglobin, mean corpuscular hemoglobin concentration, red cell distribution width, or mean platelet volume, and no significant changes were noted in blood chemistries (data not shown). Collectively, these results suggested that i.d. administration of multiple vaccines produced no adverse reactions, as determined by these assays.

### Robust antibody response to individual antigens

**Figure 2:**
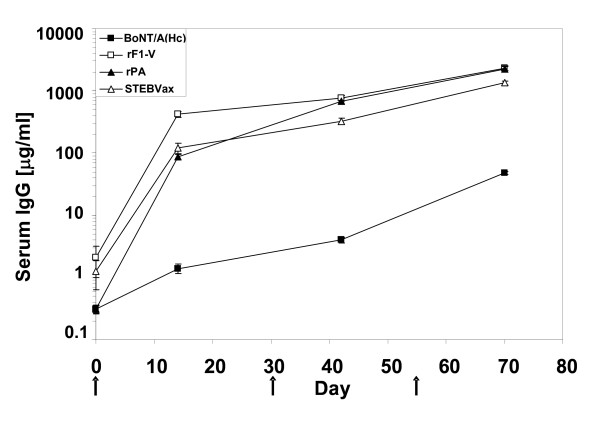
**Concurrent intradermal administration of four independent vaccines resulted in rapid seroconversion of specific IgG**. Mean ± SD (triplicate determinations) of antigen-specific IgG for all vaccinated animals. ■ BoNT/A(H_c_) vaccine, □ rF1-V vaccine, △ STEBVax, ▲ rPA vaccine. The arrows indicate the days of vaccine administration.

We next examined antibody responses to assess biological compatibility of the vaccines after i.d. administration. Sera were collected after each vaccination and antigen-specific antibodies were measured. All vaccines induced a significant increase in specific IgG compared to control by 14 days after the primary vaccine administration (Table 1). Further enhancement of the immune response to each vaccine was observed with each subsequent vaccination (Fig. 2). The final recorded antibody levels for BoNT/A(H_c_), rPA and STEBVax were similar to previous values for animals receiving individual i.m. vaccinations [11,13,17,19] and F1-V responses were the highest. Serum levels of BoNT/A-specific antibody were lowest compared to all other antibodies except controls, likely as a result of the small amount of BoNT/A(H_c_) used for vaccinations. Levels of antigen-specific IgM against all antigens were significantly elevated compared to controls 2 weeks after the final vaccine administrations (Table 1). We concluded that levels of serum antibodies against each vaccine were not altered by concurrent i.d. injection to sites that were in close proximity to each other.

**Table 1:** Robust serum antibody response to simultaneous intradermal vaccination

				Antibody concentration (μg/ml) mean ± SD			
				
				Vaccine			
				
Isotype	Day	Treatment		BoNT/A(H _c_)	rF1-V	rPA	STEBVax
IgM	70	Control	(n = 8)	3.07+/-0.87	2.99+/-1.47	6.31+/-3.16	4.76+/-3.62
	70	Vaccinated	(n = 24)	5.47+/-2.20	11.2+/-4.04	13.7+/-9.28	9.07+/-2.74
		p-value*		**0.0001**	**< 0.0001**	**0.002**	**0.012**

IgG	14	Control	(n = 8)	0.31+/-0.15	2.1+/-3.1	0.31+/-0.12	1.25+/-1.76
	14	Vaccinated	(n = 24)	1.4+/-1.1	421+/-196	86+/-46	121+/-109
		**p-value**		**< 0.0001**	**< 0.0001**	**< 0.0001**	**< 0.0001**
	
	42	Control	(n = 8)	0.28+/-0.22	1.95+/-0.98	2.2+/-1.4	1.23+/-0.91
	42	Vaccinated	(n = 24)	4+/-2.1	767+/-382	689+/-397	323+/-187
		**p-value**		**< 0.0001**	**< 0.0001**	**< 0.0001**	**< 0.0001**
	
	70	Control	(n = 8)	0.65+/-0.37	1.05+/-1.08	0.91+/-0.44	1.93+/-1.25
	70	Vaccinated	(n = 24)	48+/-13	2331+/-303	2245+/-1224	1340+/-215
		**p-value**		**< 0.0001**	**< 0.0001**	**< 0.0001**	**< 0.0001**

*Significance of mean serum IgM and IgG concentrations for control and vaccinated animals were compared using Student's t-test.

### Neutralizing antibody responses

**Figure 3:**
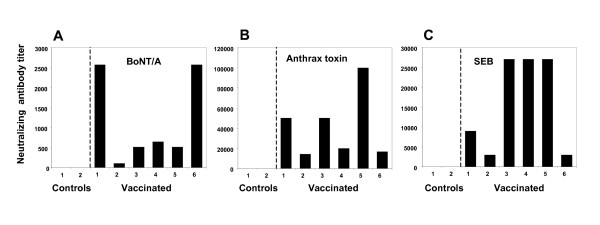
**Potent neutralizing antibody responses of rhesus macaques receiving concurrent intradermal administrations of four independent vaccines**. A. Neutralizing antibody titers for animals in: A. botulinum neurotoxin type A challenge group. B. anthrax challenge group. C. SEB challenge group. Individual animals vaccinated with antigens plus AH, Vaccinated 1–6; injected with AH only, Control 1–2. All disease challenges occurred one month after the final vaccination. Geometric mean titers, based on triplicate determinations.

Standard assays were previously established for determining the level of antibodies present in sera that protect the vaccinated host from SEB-toxic shock, botulism, and anthrax. These neutralizing antibody assays provided an additional parameter for predicting the outcome of exposure to each agent of disease. The BoNT/A neutralizing antibody titers were determined as the reciprocal of the serum dilution that protected 50% of the mice from challenge with 10 LD_50 _of toxin. Serum from vaccinated primates protected CD-1 mice challenged with BoNT/A (Fig. 3A); serum from control animals was not protective. Antibodies that neutralized *B. anthracis *were present in all vaccinated animals, but not in controls, as determined by measuring inhibition of J774 cell lysis after exposure to anthrax lethal toxin (Fig. 3B). Additionally, serum from vaccinated animals prevented SEB-induced proliferation of human peripheral blood mononuclear cells after addition of the toxin to culture (Fig. 3C). We could not determine the titers of neutralizing antibody against plague because there were no previously validated assays available for the rhesus monkey that permitted correlation of antibody titer with protection from disease.

### Protection from multiple bacterial and toxin-mediated diseases

**Figure 4:**
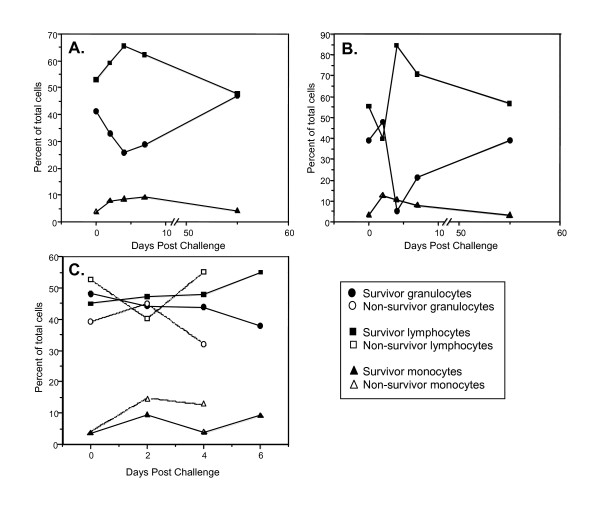
**Vaccination resulted in rapid recovery of white blood cell populations following disease challenge**. All disease challenges occurred one month after the final vaccination. Peripheral arterial blood was drawn at various time points postchallenge and analyzed for changes in cellular composition. A. Botulinum neurotoxin type A; B. Staphylococcal enterotoxin B. C. *B. anthracis *(Ames) spores.

The results up to this point demonstrated robust antibody responses to all vaccines and these titers were similar or identical to previous studies using monovalent i.m. vaccinations [11,13,17,19]. Therefore, we next evaluated protection of vaccinated animals from disease. The rhesus macaques were healthy with no overt signs of disease or pathology before challenge. The total white blood cell counts and distribution of granulocytes, monocytes, and lymphocytes remained within normal range throughout the study for all vaccinated and control animals prior to disease challenge, indicating minimal systemic inflammatory responses to the multiple vaccines or method of administration (Fig. 4A–C). These data were in accordance with the general blood chemistry profiles (described above). This cellular data was collected to follow any potential toxicity resulting from the experimental method and to address the outcome of vaccinations on the inflammatory response occurring during the early stage of disease onset. The animals were divided into four separate challenge groups consisting of two controls and six vaccinated rhesus macaques. Each group was challenged by aerosol with either BoNT/A, SEB, or B. anthracis (Ames) spores and monitored for up to 2 months post-challenge. All disease challenges occurred one month after the final vaccination. Slight to moderate fluctuations in the distribution of white cell populations were noted for all animals within the first 48 h following challenge with toxin or bacteria (Fig. 4), perhaps due to a generalized inflammatory response to aerosol challenge. Efficacy was evaluated by comparing the treated group to the control group for each agent consisting of the 2 experimental controls and 15 historical controls. Uniform lethality has been observed in more than 15 untreated control rhesus exposed to the same strain and route of each agent used in the experiment (unpublished observations). Results indicated that the percentage of animals surviving in each treatment group (6/6 or 100%) was significantly higher than the percentage of animals surviving in each pooled control group (0/17 or 0%), p < 0.0001. Further details concerning each disease challenge are described below.

All vaccinated animals receiving BoNT/A (65 × LD_50 _average) survived (Table 2) and exhibited no outward clinical signs of botulism. Both control animals survived for only 2 days after challenge and necropsy findings were suggestive of death due to BoNT/A intoxication, although no specific post-mortem lesions are induced by BoNT/A. These findings included aspiration of foodstuff into the trachea and lungs due to dysphagia secondary to cranial nerve paralysis after exposure to the toxin. White blood cell counts of the vaccinated animals were only slightly affected by challenge. However, the average percentage of lymphocytes and monocytes increased, while granulocytes decreased until about 4 days post-challenge (Fig. 4A). Each cell population returned to normal pre-challenge levels by day 55 post-challenge.

**Table 2:** Simultaneous intradermal vaccination with four independent vaccines protected Rhesus macaques from fatal infectious or toxin-mediated disease

	Bot/A Challenge*		Spore Challenge		SEB Challenge	
			
	Dose (LD50s)	Survival**	Dose (LD50s)	Survival	Dose (LD50s)	Survival
Control 1	57	-	507	-	33.5	-
Control 2	100	-	412	-	18.0	-

Vaccinated 1	50	+	257	+	26.4	+
Vaccinated 2	24	+	487	+	25.6	+
Vaccinated 3	99	+	439	+	15.8	+
Vaccinated 4	43	+	373	+	18.9	+
Vaccinated 5	82	+	275	+	19.6	+
Vaccinated 6	62	+	263	+	23.4	+

Mean+/-SD	65+/-27		377+/-101		23+/-6	

*All disease challenges occurred one month after the final vaccination.

**Efficacy was evaluated using Fishers exact test comparing the treated group to the control group for each agent consisting of 2 experimental controls and 15 historical controls. Results indicated that the percentage of animals surviving in each treatment group (6/6 or 100%) was significantly higher than the percentage of animals surviving in each pooled (experimental plus historical) control group (0/17 or 0%), p < 0.0001.

All of the vaccinated animals survived challenge with SEB (23 × LD_50 _average), showing no clinical signs of toxic shock after challenge (Table 2). In contrast, control animals survived for only 2 days after challenge. Necropsy and histopathology verified that death of the controls was consistent with toxic shock caused by SEB. Total white blood cells of the vaccinated animals did not significantly change after challenge. Similar to profiles of vaccinated animals surviving botulism, the percentage of lymphocytes and monocytes increased while the percentage of granulocytes decreased until about day 4 (Fig. 4B). The percentage of each cell type then returned to prechallenge levels by day 55 postchallenge.

Control animals exposed to *B. anthracis *spores (377 × LD_50_) survived 4 days after challenge and death corresponded with an increase in bacteremia detectable by day 4. The control animals exhibited increased blood monocytes (2 d) and granulocytes (4 d), while lymphocytes decreased by 4 days after challenge. Necropsy and histopathology verified that death was consistent with anthrax. All spore-challenged animals that were vaccinated survived with no disease symptoms (Table 2), and no significant changes in granulocytes, lymphocytes, or monocytes were observed (Fig. 4C).

## Discussion

Our data demonstrates that i.d. vaccination of multiple antigens by a method that physically separates each component circumvents the primary physical, chemical, and biological incompatibilities that are common to combination vaccines prepared by mixing before administration. Our results with four unique diseases suggested that we did not reach a biological limit to the number of vaccines that can be administered at one time and that there was no apparent "vaccine overload" [1]. Any injection site trauma appeared to be minor due to the minute size of the needles used, consistent with a previous clinical study [3]. We observed small blebs on the skin of rhesus macaques immediately after vaccination, resulting from the fluid injected, while these sites were barely perceptible by the end of the study and surrounding tissues returned to normal by 3 months. All of the vaccines we examined induced significant levels of serum antibodies (IgM, IgG), equivalent to historic data and neutralizing antibody titers were observed for anthrax, BoNT/A, and toxic shock vaccines. All vaccinated rhesus macaques were protected from an otherwise lethal anthrax, botulism and staphylococcal toxic shock. Our results indicated that the percentage of animals surviving in each treatment group (6/6 or 100%) was significantly higher than the percentage of animals surviving in each pooled control group (0/17 or 0%), p < 0.0001. Collectively, these results indicate that the vaccines were biocompatible by i.d. administration and physical separation. Seroconversion also occurred after the primary dose for each vaccine, though it is not clear if this was dependent on the method of delivery. The rF1-V vaccine was previously shown to be protective against plague in mice [18,19] and this was confirmed with the vaccine used in our study (data not shown). Yet, there is a paucity of published data for efficacy of vaccines based on the LcrV and CaF1 antigens in non-human primates. Antibody levels specific for rF1-V were the highest among all of the vaccinated animals, suggesting that the potency of this vaccine was maintained. Cellular immunity, not addressed in our study, may also be important for protection from plague [22]. We observed that the minor perturbations of blood cell counts occurring within days of challenge returned to normal for all vaccinated animals.

Notably, the significance of our results should be considered in light of the general benefits of vaccination to society. For example, there are substantial cost savings to the individual and to the public resulting from protection against the 11 diseases preventable by the current routine childhood vaccination schedule [23]. However, there are currently 28 recommended vaccines for children and adults, plus annual influenza vaccinations. Additional vaccines are planned for protection from the nine category A and numerous B-C agents on the Centers for Disease Control and Prevention (CDC) select agent list. Therefore, developing a reasonable vaccination schedule that assures patient compliance is a significant public health objective. Combination vaccines offer one solution, yet these are often difficult and costly to develop due to product incompatibilities that may not be apparent during development of individual component antigens.

Previous studies demonstrated that vaccine efficacy was improved by targeting the dermis of the skin for delivery [4,5,20,24-26], resulting in dose sparing by a mechanism that is not clearly established. In our study, immune responses to vaccines administered i.d. were not isolated to the skin, though an enhancement of regional tissue immunity may also have been possible. We observed that the vaccines were internalized by dermal antigen-presenting cells and transported to the draining axillary lymph nodes. It is unclear if physiological transport of the vaccines delivered i.d. differs substantially from i.m. vaccination. Regardless of the mechanism, it should also be possible to increase the total number of vaccines that can be administered to a small dermal site by lowering the delivery volume for individual components because reduced amounts of antigen are required for i.d. vaccination.

## Conclusion

The physical separation of vaccines both in the syringe and at the site of administration did not adversely affect the biological activity of any component vaccine. Further, the vaccination method we describe may be scalable to include a greater number of antigens, while avoiding the physical and chemical incompatibilities encountered by combining multiple vaccines together in one product. Our results demonstrate that intradermal delivery of multiple vaccine preparations may provide a practical alternative to traditional combination vaccines and complicated administration schedules.

## Abbreviations

AH: aluminum hydroxide adjuvant; BoNT/A: botulinum neurotoxin type A; BoNT/A(H_c_): recombinant botulinum neurotoxin type A heavy chain; i.d.: intradermal; rF1-V: recombinant fusion protein of the F1 and V antigens; rPA: recombinant protective antigen; STEBVax: recombinant staphylococcal enterotoxin B vaccine; SEB: staphylococcal enterotoxin B

## Competing interests

Jason B. Alarcon and John A. Mikszta are employed by Becton Dickinson Technologies, the manufacturer of the micro-needle device used in this study. All other authors declare no potential conflicts of interest

## Authors' contributions

GLM participated in the design of the study, performed the vaccinations, analyzed data and drafted the manuscript. RFT performed the botulism studies and analyzed the data. BKP performed bacterial challenge studies and analyzed the data. PLW participated in the design of the study and analyzed data from the bacterial challenges. JC carried out the necropsy and histology studies of all animals. LSM contributed the botulinum toxin vaccine and analyzed data from the botulism study. JBA performed the vaccinations and analyzed data. JAM participated in the design of the study, developed the vaccination device and analyzed data. RGU conceived of the study, participated in its design and coordination, and drafted the manuscript.
